# Controlled balloon false lumen obliteration for the endovascular management of chronic dissection in the descending thoracic aorta

**DOI:** 10.1016/j.xjtc.2023.01.010

**Published:** 2023-01-23

**Authors:** A. Claire Watkins, Shernaz Dossabhoy, Alex R. Dalal, Aleena Yasin, Matthew Leipzig, Benjamin Colvard, Jason T. Lee, Michael D. Dake

**Affiliations:** a Department of Cardiothoracic Surgery, Stanford University, Stanford, Calif; b Division of Vascular Surgery, Stanford University, Stanford, Calif; c Division of Vascular Surgery and Endovascular Therapy, University Hospitals Cleveland Medical Center, Cleveland, Ohio; d University of Arizona Health Sciences, Tucson, Ariz

**Keywords:** Aortic dissection, Stent grafting, Thoracic endovascular aortic repair

## Abstract

**Objective:**

Retrograde false lumen perfusion has limited the utility of aortic stent grafting for chronic aortic dissection. It is unknown whether balloon septal rupture can improve the outcomes for endovascular management of chronic aortic dissection.

**Methods:**

Included patients underwent false lumen obliteration and creation of a single-lumen aortic landing zone using balloon aortoplasty during thoracic endovascular aortic repair. The distal thoracic stent graft was sized to the total aortic lumen diameter, and septal rupture was performed within the stent graft with a compliant balloon in the region 5 cm proximal to the distal fabric edge. Clinical and radiographic outcomes are reported.

**Results:**

Forty patients, with an average age 56 years, underwent thoracic endovascular aortic repair with septal rupture. Seventeen patients (43%) had chronic type B dissections, 17 of 40 patients (43%) had residual type A dissections, and 6 of 40 patients (15%) had acute type B dissections. Nine cases were emergency, complicated by rupture or malperfusion. Perioperative complications included 1 death (2.5%) due to rupture of the descending thoracic aorta and 2 (5%) instances each of stroke (neither permanent) and spinal cord ischemia (1 permanent). Two (5%) stent graft–induced new injuries were seen. Average postoperative computed tomography follow-up was 1.4 years. Thirteen patients (33%) had a decrease in aortic size, 25 of 39 patients (64%) were stable, and 1 of 39 patients (2.6%) had an increased aortic size. Partial and complete false lumen thrombosis were achieved in 10 of 39 patients (26%) and 29 of 39 patients (74%), respectively. Midterm aortic-related survival was 97.5% at an average of 1.6 years.

**Conclusions:**

Controlled balloon septal rupture offers an effective endovascular method to treat aortic dissection in the distal thoracic aorta.

## Graphical abstract

**Figure:**
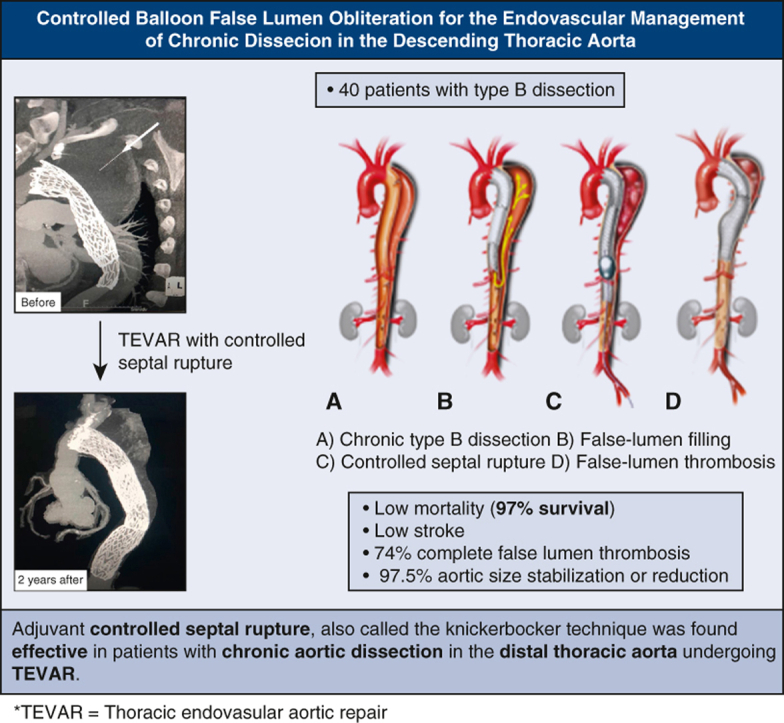

**Figure:**
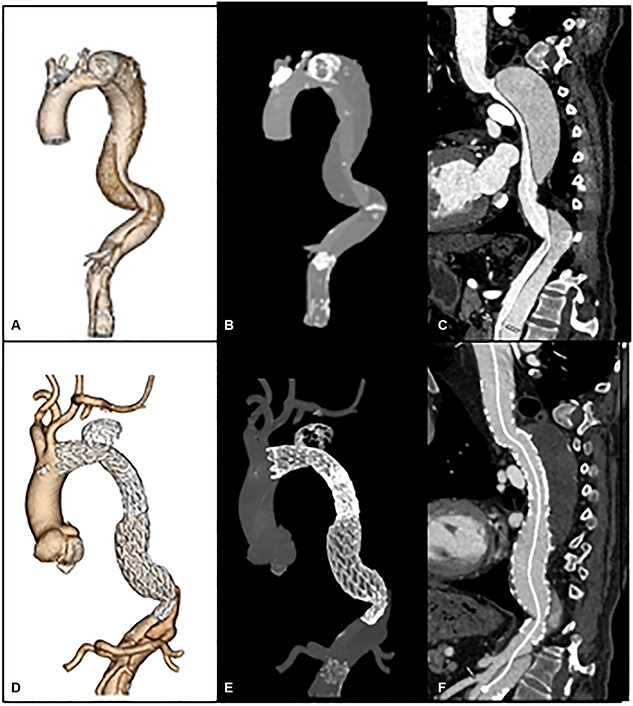
Preoperative computed tomography angiography imaging of a chronic type B dissection with aneurysm.

**Central Message**

Balloon-induced rupture of the aortic dissection membrane after endograft placement for chronic dissection prevents retrograde FL perfusion. Further research should consider this technique.

**Perspective**

Endovascular management of aortic dissection has evolved significantly since 1999. Results for chronic dissection have been limited by retrograde FL perfusion. False lumen closure techniques have demonstrated improved outcomes. This study reports promising perioperative and midterm outcomes of balloon septal rupture during TEVAR.

Chronic type B aortic dissection (cTBAD) with aneurysm traditionally has had suboptimal results with endovascular therapy, with only 40% of patients achieving complete false lumen (FL) thrombosis.^1^^,^^2^ Thoracic endovascular aortic repair (TEVAR) for acute type B is considered safer than medical management or open surgical repair with decreased morbidity and mortality.^3^^,^^4^ However, debate exists regarding the efficacy of TEVAR as the sole intervention in cTBAD. TEVAR serves to cover the primary tear in cTBAD and depressurize and promote thrombosis of the FL, but does not prevent retrograde FL filling through distal fenestrations. As type B aortic dissection becomes chronic, the intimal dissection flap becomes thicker and more fibrotic, reducing capacity for effective aortic remodeling after TEVAR. To prevent long-term aortic complications and death, it becomes imperative to not only cover the proximal entry tear but also prevent FL retrograde flow. Several techniques to obstruct the FL have been described. Balloon septal rupture uses balloon aortoplasy to induce fracture of the dissection septum, relaminate the aortic layers, create a single aortic lumen, and block retrograde FL perfusion. The aim of this study is to review the clinical and radiographic outcomes of endovascular management of cTBAD using controlled balloon septal rupture.

## Materials and Methods

### Patient Cohort

All elements of this study were approved by the Institutional Review Board at Stanford University (Institutional Review Board #25673, 7/19/22) with a waiver of individual patient consent. Forty patients undergoing TEVAR with controlled balloon septal rupture technique for chronic aortic dissection between 2017 and 2022 were reviewed retrospectively. Clinical and radiographic outcomes were collected from the electronic medical records.

### Inclusion and Exclusion Criteria

To be considered for adjuvant balloon septal rupture, patient anatomy had to meet the following criteria: (1) an adequate proximal landing zone and (2) a segment of distal descending thoracic aorta that was less than 45 mm in diameter, greater than 3 cm in length, and greater than 5 cm proximal to the origin of the celiac artery. If not, patients were excluded. All cases used the Gore conformable-TAG stent graft, the largest size of which is 45 mm, accounting for the diameter limitation. The location greater than 5-cm length proximal to the celiac artery criteria allows the stent to make a gradual taper distal to the area of ballooning, helping to avoid aortic injury. Additional exclusions included inadequate iliofemoral access.

### Operative Technique

All TEVARs were performed in a hybrid operating room under general anesthesia. Lumbar drains were used at the discretion of the surgeon with a substantial use in those with greater than 250 mm of aortic coverage, prior descending or abdominal aortic surgery, or missing vertebral or hypogastric perfusion. All cases used the Gore conformable-TAG stent graft (WL Gore & Associates).

**Figure 1:**
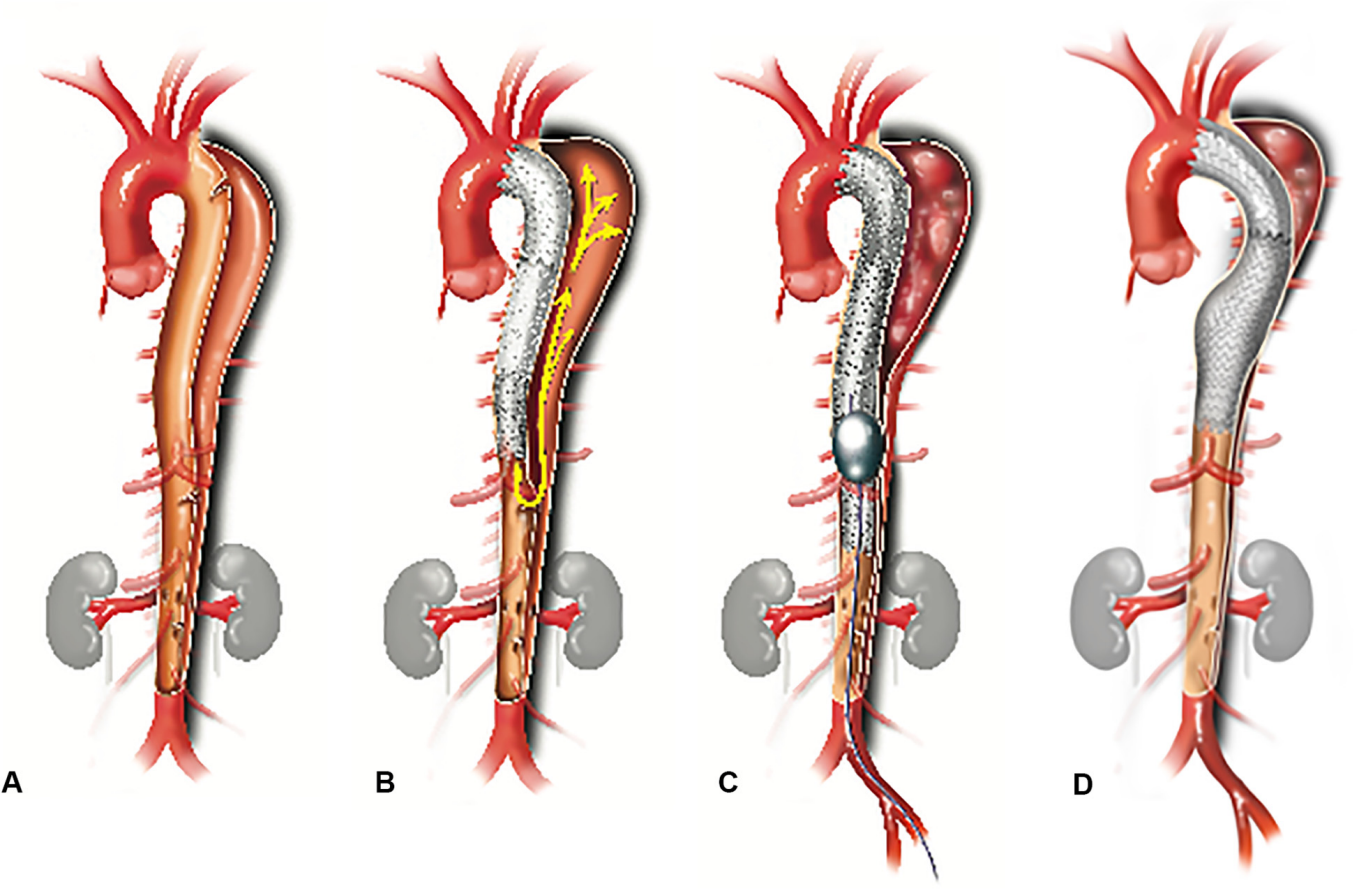
A, Type B aortic dissection. B, Type B aortic dissection with traditional TEVAR. C, Controlled balloon septal rupture after TEVAR done in the planned distal landing zone but approximately 5 cm from the distal end of the stent graft. D, Completed TEVAR with balloon septal rupture for FL closure in cTBAD.

First, TEVAR was performed originating proximal to the primary entry tear and terminating at the level of the celiac artery. The ideal proximal landing zone goal was 2 cm of healthy aorta or surgical graft with a goal of 10% to 15% major aortic diameter oversizing. Second, a Gore Trilobe balloon is then gradually inflated in the distal landing zone and expanded beyond the device's original contour as seen on fluoroscopy. Upon balloon deflation, the endograft assumed the typical “Knickerbocker” configuration with a focal bulge at the site of the septal disruption, where the endograft then creates a circumferential seal around the outer wall of the newly formed single aortic channel. Distal landing zone sizing goal was 1-to-1, without oversizing. The stent graft extends at least 5 cm beyond the area of intended controlled balloon septal rupture or fracture, creating a taper in the distal stent graft (Figure 1 and Video 1). Pre- and postballooning aortograms were used to evaluate for type Ib endoleaks and complications. Evidence of FL filling on completion angiogram and evidence of stent-graft underexpansion on fluoroscopy were used to determine degree of ballooning. A conservative approach was generally taken given the relative risks of endoleak versus aortic injury. Technical success was defined as no type I or III endoleak or vascular injury at the end of the operation.

### Follow-up and Analysis

**Figure 2:**
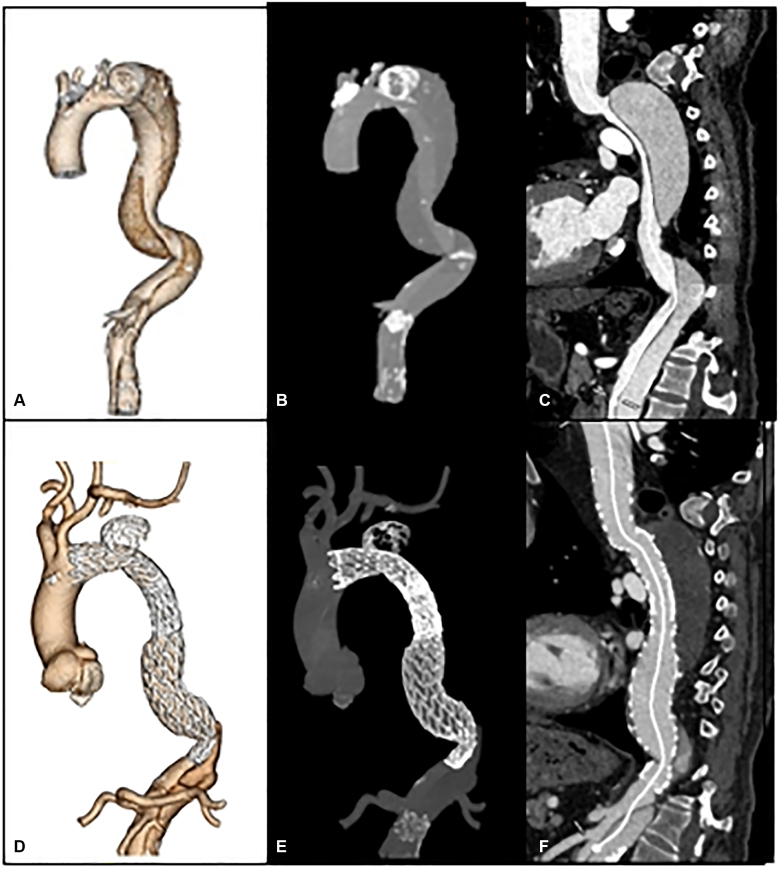
A-C, Preoperative computed tomography angiography imaging of a chronic type B dissection with aneurysm. D-F, Postoperative imaging after TEVAR with balloon septal rupture, demonstrating occlusion of retrograde FL filling at the level of ballooning.

All patients received computed tomography angiography before TEVAR and immediately postoperatively, and at 1, 6, and 12 months to identify endoleaks and thoracic aortic remodeling (Figure 2). Imaging acquisition technique included noncontrast, arterial and delayed phases, electrocardiogram gating, and sub-millimeter slices. Radiographic analyses were done by the aortic surgeon and a cardiovascular radiologist. At each follow-up, transaortic diameters were measured at the same anatomic location and compared with the patient's immediate postoperative imaging. Aortic growth or shrinkage was defined as more than 2-mm change in aortic size. False lumen (FL) patency was determined by contrast in the FL on arterial or stent delay images in the stented portion of the aorta. FL thrombosis was characterized as complete, partial, or absent. Positive remodeling was defined as stabilization or reduction of aortic size. Clinical follow-up was done through telehealth, virtual, or in-person aortic surveillance clinic appointments similarly at 1, 6, and 12 months. Patients were assessed for late complications and progression of aortic disease.

### Statistical Methods

Statistical analysis was performed in RStudio 1.4.1717 (RStudio, Inc). Continuous variables are shown as a mean and standard deviation. Categorical variables are shown as percentages. Patient survival was assessed with Kaplan–Meier analysis. Kaplan–Meier estimates were generated using the survminer R package.

## Results

### Demographics

Forty patients, with an average age of 56 ± 9 years, underwent TEVAR with the balloon septal rupture technique between 2017 and 2022 (Table 1). The majority of patients were male (95%). Hypertension was the most common risk factor and was present in all of the patients, followed by hyperlipidemia (95%). Other comorbidities such as smoking history (55%), chronic kidney disease (43%), and cerebral arterial disease (35%) were not uncommon. During the 6-year study period, 17 patients presented with chronic type B dissections, 17 patients presented with residual type A dissection, and 6 patients presented with acute type B dissections. Nine patients (23%) presented with rupture or malperfusion. Twenty-seven patients (68%) had undergone prior aortic surgeries (82% open and 19% endovascular). Most cases were due to degenerative atherosclerotic disease and 2 patients had a connective tissue disorder. The mean time between the initial diagnosis and intervention was 3.3 years (1221 ± 1670 days).

**Table 1:** Preoperative and operative characteristics

Characteristic	N = 40 (%)
Age, y	56 ± 9
Sex, male	38 (95)
Pathology	
Acute type B	6 (15)
Chronic type B	17 (43)
Residual type A	17 (43)
Prior aortic surgery	27 (68)
Open	22 (82)
Endovascular	5 (19)
Rupture	5 (12)
Malperfusion	4 (10)
Shock	4 (10)
Acidosis	4 (10)
Connective tissue disease	2 (5)
Hypertension	40 (100)
Hyperlipidemia	33 (83)
Tobacco use	
Never	18 (45)
Current	9 (23)
Former	13 (33)
Diabetes	2 (5)
Chronic obstructive pulmonary disease	3 (7.5)
Coronary artery disease	4 (10)
Congestive heart failure	4 (10)
Ejection fraction	58 ± 8
Peripheral artery disease	4 (10)
Cerebral artery disease	14 (35)
Chronic kidney disease	17 (43)
Hemodialysis	0
Atrial fibrillation	4 (10)
Anticoagulation	6 (15)
Antiplatelet medication	19 (48)
Immunosuppression	1 (2.5)
Proximal aortic landing zone	
2	20 (50)
3	20 (50)
Lumbar drain	27 (68)
Side branch stenting	5 (13)

### Operative Characteristics

During TEVAR, 5 patients required additional stenting: 2 left common carotid artery, 1 superior mesenteric artery, 1 iliac artery, and 1 renal artery (Table 1). Four of these were planned, and 1 left carotid stent was unplanned. Prophylactic cerebrospinal fluid drainage was used in 27 patients (68%) for prevention of spinal cord ischemia (SCI). The proximal landing zone was in zone 2 of the aortic arch in 20 patients (50%) and in zone 3 in 20 patients (50%). Two cases were done as the first of a planned 2-stage thoracoabdominal repair. One case was done as a hybrid approach at the time of aortic arch replacement due to unique anatomy.

### Operative Outcomes

Perioperative mortality was 2.5% (n = 1) (Table 2). The single patient in this cohort died secondary to aortic rupture at the distal landing zone. There was a total of 2 (5%) cerebrovascular accidents reported, neither of which caused permanent deficit. Two patients (5%) had SCI. One was treated with a lumbar drain and completely resolved without any sequelae. The other patient was discharged with residual weakness. Five patients (13%) had in-hospital acute kidney insufficiency. Only 1 patient (2.5%) required postdischarge dialysis. One of the temporary strokes, the case of permanent SCI and the case of long-term dialysis, all occurred in the same patient with acute type B dissection presenting with profound malperfusion.

**Table 2:** Operative outcomes (<30 d or in-hospital)

Outcome	N = 40 (%)
Death	1 (2.5)
Aortic-related death	1 (2.5)
Stroke	2 (5)
Permanent	0
SCI	2 (5)
Permanent	1 (2.5)
Lumbar drain complication (N = 27)	4 (15)
Vascular access complication	1 (2.5)
Acute kidney injury	5 (13)
Dialysis at discharge	1 (2.5)
Unplanned reintervention	5 (13)
Open	0
Endovascular	5 (13)
Length of stay	11 ± 11

*SCI*, Spinal cord ischemia.

Among the 27 patients with preoperative lumbar drain placement, 4 (15%) had drain-related complications (1 epidural hematoma, 1 minor subarachnoid hemorrhage, and 2 cerebrospinal fluid leaks).

There were no instances of retrograde type A dissection due to TEVAR. There were 2 cases of balloon or stent graft–induced new aortic injury (SINE). As reported above, 1 led to rupture. The second led to radiographic, but not clinical, superior mesenteric artery (SMA) malperfusion with subsequent SMA stenting. The average length of stay was 11 ± 12 days.

### Radiographic Outcomes

Radiographic follow-up was on average 1.24 years (452 ± 402 days). Sixteen patients (41%) had endoleaks within 30 days of surgery (Table 3). There was 1 type Ia, 8 type Ib, 7 type II, and no type III endoleaks. Among the 11 (69%) early endoleaks that were treated conservatively, 4 have resolved during follow-up, 4 are ongoing with aneurysm size stability, and the results of 2 are unknown.

**Table 3:** Endoleak characteristics

Early (<30 d) N = 16					
Type	N	Management		Outcome	Aortic size
Ia	1	Medical		Ongoing	Stable
Ib	8				
	6	Medical			
			3	Resolved	Stable or smaller
			2	Ongoing	Stable
			1	Unknown	Unknown
	2	Interventional	2	Resolved	Stable
II	7				
	4	Medical			
			1	Resolved	Stable or smaller
			2	Ongoing	Stable
			1	Unknown	Unknown
	3	Interventional			
			3	Resolved	Stable or smaller

Late (>30 d) N = 3					
Type	N	Management		Outcome	Aortic size
II	3	Medical	2	Resolved	Smaller
			1	Ongoing	Larger

There were 3 new type II endoleaks occurring after 30 days. In 2 patients, the type II that was managed conservatively resolved and aneurysm shrinking was seen. There was 1 case of a type II endoleak developing 1 year after TEVAR and leading to slow aneurysm growth. This was not treated further because the patient had chronic end-stage respiratory failure and ultimately died of ventilator-associated pneumonia 1.5 years after TEVAR.

Complete FL thrombosis was achieved in 74% and partial FL thrombosis in 26% (Table 4). Among the 10 with partial FL thrombosis, aortic size was stable or smaller in 90%. Aortic size was stable in 64% (25/39) and decreased in one-third (33%) of the patients, whereas aortic size increased in 1 previously described patient (2.6%). Favorable aortic remodeling was achieved in 97% of patients (38/39).

**Table 4:** Midterm clinical and radiographic outcomes (>30 and out of hospital)

Outcome	N = 39 (%)
Deaths	1 (2.6)
Aortic related	0
Stroke	0
Permanent	0
Spinal cord ischemic	0
Permanent	0
Aortic rupture	0
Unplanned reintervention	7 (18)
Open	4 (57)
Endovascular	3 (43)
False lumen thrombosis	
None	0
Partial	10 (26)
Complete	29 (74)
Aortic size progression	
Decrease	13 (33)
Stable	25 (64)
Increase	1 (2.6)
Length of follow-up (d)	
Clinical	662 ± 502
Radiographic	452 ± 402

### Unplanned Aortic Reinterventions

There were 12 unplanned aortic reinterventions in 10 patients (32%); 5 were perioperative, and 7 were long term. Among the early reinterventions, all were endovascular. Two were performed in the setting of acute type B dissection with malperfusion. Two were performed for type 2 endoleak originating at the left subclavian artery. One reintervention was for SINE and radiographic SMA malperfusion, as mentioned above.

Among the late reinterventions, 3 were endovascular and 4 were surgical. One of the endovascular reinterventions was a successful re-ballooning of the distal landing zone to close a type Ib endoleak. Three of the open surgical aortic repairs were for distal disease progression, unrelated to the initial repair. The remaining 3 late reinterventions were in a single patient who underwent TEVAR extension and re-ballooning for a type Ib endoleak and aortic growth. This reintervention was complicated by SINE causing malperfusion and requiring surgical femoral artery repair and SMA stenting. Among both early and late reinterventions, there were no anatomic features, including aortic size, associated with reintervention. No reinterventions were associated with mortality. Among those requiring reintervention, the only mortality was in a patient with chronic respiratory failure who died of ventilator-associated pneumonia 1.5 years after TEVAR and reintervention.

### Midterm Outcomes

**Figure 3:**
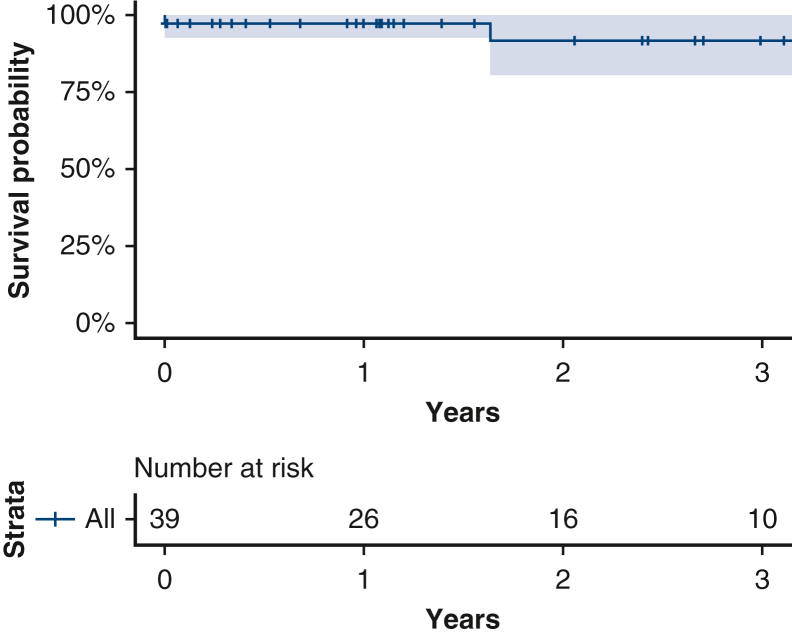
Survival after TEVAR with controlled balloon septal rupture for type B aortic dissection (95% confidence limits).

There was no midterm aortic-related mortality. Overall survival was 95% (38/40), and aortic-related survival was 97.5% (39/40) during the mean clinical follow-up duration of 1.81 years (662 ± 502). The Kaplan–Meier survival plot is presented in Figure 3. Survival was 98% and 95% at 1 and 2 years, respectively. After the operative period, there was no incidence of major neurological complications including stroke or paraplegia.

## Discussion

**Figure 4:**
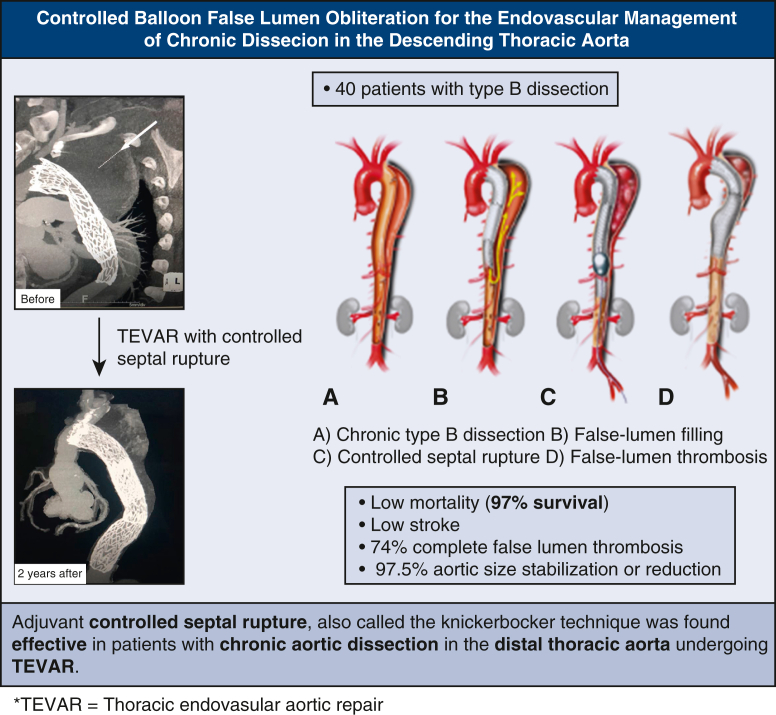
Graphical Abstract summarizing results. *TEVAR*, Thoracic endovascular aortic repair.

Traditionally, type B dissection treated with only coverage of the primary entry tear often leads to persistent retrograde filling of the FL and aortic growth. As the dissection becomes more chronic, dissection septal compliance decreases, as does the likelihood of complete FL thrombosis and aortic stabilization with TEVAR. Only 50% of patients treated with TEVAR alone for chronic type B dissection achieve complete FL thrombosis and only 65% demonstrate beneficial aortic remodeling,^2^ with a significant mortality related to late-aortic events.^4^ Various methods to prevent distal FL perfusion have been described, including embolization, longitudinal fenestration, and balloon septal fracture. We report the clinical and radiographic outcomes of 40 patients undergoing balloon septal fracture to obliterate the distal FL in type B aortic dissection (Figure 4). Highlights of our reported results include a midterm, aortic-related mortality of 2.5% in a considerably high-risk patient cohort, with many being refused for open surgical repair. Additionally, neurologic complications were low. Compared with traditional TEVAR for chronic type B dissection, as well as open surgical repair, the frequency of neurologic complications, mainly stroke and permanent SCI, were similar or reduced.^4^ Although this study had 1 mortality and 2 malperfusion events due to aortic ballooning, it also had a 24% increase in complete FL thrombosis and 32% increase in favorable remodeling compared with that in the literature,^2^^,^^4^ highlighting the high-risk high reward nature of this technique. Similar results of this technique have been reported by Rohlffs and colleagues^5^ and Levack and colleagues.^6^ The rate of lumbar drain complications (15%) was higher than typically reported in the literature, which questions the benefit of our relatively high lumbar drain use (68%).

The rate of early endoleak in this study was relatively high at 41% (16/39). Our practice of high-quality computed tomography angiography before discharge likely contributed to an increased detection rate. The majority, 69% (11/16), of endoleaks were treated medically. Of all early endoleaks available for follow-up (14/16), none were associated with aortic expansion. Among all early and late endoleaks, only 1 (2.5%) was associated with adverse aortic growth. Rates of both early and late unplanned aortic reinterventions were similar to that published in the literature. Among the reinterventions in this study, 2 endoleaks from the left subclavian could have been avoided with more aggressive oversizing during embolization and 2 instances of malperfusion after shift in the dissection flap could have been avoided with prewiring of side branches originating from the FL. Although many consider endoleak and subsequent aortic reintervention to be the Achilles' heel of TEVAR, it should be noted that there were no mortalities due to endoleak or reintervention. Also, the 97.5% rate of beneficial aortic remodeling is a significant improvement compared with historical results. This is consistent with other reports in the field suggesting reintervention does not correlate with decreased morbidity or mortality.^7^^,^^8^ Perhaps the most concerning outcome in this study is the single incidence of aortic rupture and the 2 cases of SINE causing malperfusion. Traditional teaching in aortic endografting prohibits ballooning of acute aortic dissection due to the risk of aortic injury. In a minority of patients in the study, balloon-assisted FL closure was necessary to contain hemodynamically unstable aortic rupture. Otherwise, ballooning of an acute dissection should be avoided because of increased aortic fragility that could lead to rupture or SINE. Others have reported SINE due to TEVAR with balloon septal rupture causing malperfusion as well.^6^ All reported incidences have been successfully managed endovascularly, but there is clearly some unpredictability in the dynamics and compliance of the dissection flap with ballooning. We have found that 5 cm or greater of taper stent graft distal to the ballooned distal landing zone is critical to avoid SINE. In our 1 case of aortic rupture, the stent graft in the distal landing zone was 12% oversized, likely contributing to the complication. The 1:1 sizing in the distal landing zone and area of ballooning cannot be overemphasized to ensure the safety of this technique. Although an overall operative survival and positive aortic remodeling are generally considered acceptable, further research is required to determine the extent of the periprocedural risk.

Techniques to obliterate the FL of aortic dissection in the descending thoracic aorta have been increasingly reported since 2003. Various devices have been used to embolize the FL, including coils, glue, Amplatzer plugs, and the custom devices (“Candy plug”).^9^ Less commonly reported, a longitudinal or “cheesewire” fenestration can be performed to create a single lumen landing zone for TEVAR.^10^ Although similar, the STABILISE technique includes ballooning of the aorta; however, it adds an uncovered stent and balloons more than a 3- to 5-cm landing zone, often to the aortic bifurcation. It is generally done in acute dissections for malperfusion, although recently has been reported for chronic dissected aneurysm. Our study did not examine the ability of a bare-metal stent to promote favorable remodeling in the thoracoabdominal and infrarenal aorta, but focused on the ability to exclude a dissected aneurysm in the DTA. Interestingly, most STABILISE studies describe a goal oversizing of 10%. We have learned that oversizing can be dangerous when ballooning an injured aorta. Additionally, although many in the field casually refer to our described technique as the “Knickerbocker” technique, the Knickerbocker technique originally described by Kolbel^11^ uses a specifically designed tapered stent graft. A septal rupture or fracture creates the same effect using off-the-shelf products.

In this study, controlled balloon septal rupture has the advantages of a high rate of complete FL thrombosis and aortic stabilization or beneficial remodeling. However, the procedure risk may be significant with both rupture and SINE being seen in this cohort. Although all techniques to close the FL have their advantages and disadvantages, it is unknown how balloon septal rupture compares with other methods.^12^, ^13^, ^14^, ^15^, ^16^, ^17^ Additionally, success is highly predicated on patient anatomy, which must include a distal segment where the transaortic diameter is no greater than the largest available endograft diameter to facilitate complete 360^o^ wall apposition and FL closure. Therefore, not all chronic type B dissections with aneurysm will be amenable to this type of repair. It is difficult to know what percentage of all type B aneurysms could be treated this way; in our experience, it has been approximately 50%.

### Study Limitations

This study has several limitations, mostly due to a small patient cohort and limited duration of follow-up. Additionally, there is no control group for examination of this novel, endovascular technique. There are no prospective clinical trials comparing open descending aortic repair with endovascular management of chronic type B dissection, yet alone including adjuvant techniques for FL closure.

## Conclusions

Controlled balloon septal rupture as an adjuvant technique to TEVAR is a viable and successful option in treating acute complicated and chronic type B dissection endovascularly. Further research is needed to know how it compares to other FL closure techniques or open surgery.

### Conflict of Interest Statement

A.C.W. is a consultant for WL Gore and is on the Aortic Advisory Board for Artivion. All other authors reported no conflicts of interest.

The *Journal* policy requires editors and reviewers to disclose conflicts of interest and to decline handling or reviewing manuscripts for which they may have a conflict of interest. The editors and reviewers of this article have no conflicts of interest.

## Supplementary Data

Video 1 A summary of the balloon septal rupture technique, results, and implications, including intraoperative examples. Video available at: https://www.jtcvs.org/article/S2666-2507(23)00037-8/fulltext.
