# Supplementary figures and images for: Controlled balloon false lumen obliteration for the endovascular management of chronic dissection in the descending thoracic aorta
Source: JTCVS Tech. 2023 Jan 23;19:1–9. doi: 10.1016/j.xjtc.2023.01.010 (PMC10267755; doi:10.1016/j.xjtc.2023.01.010)

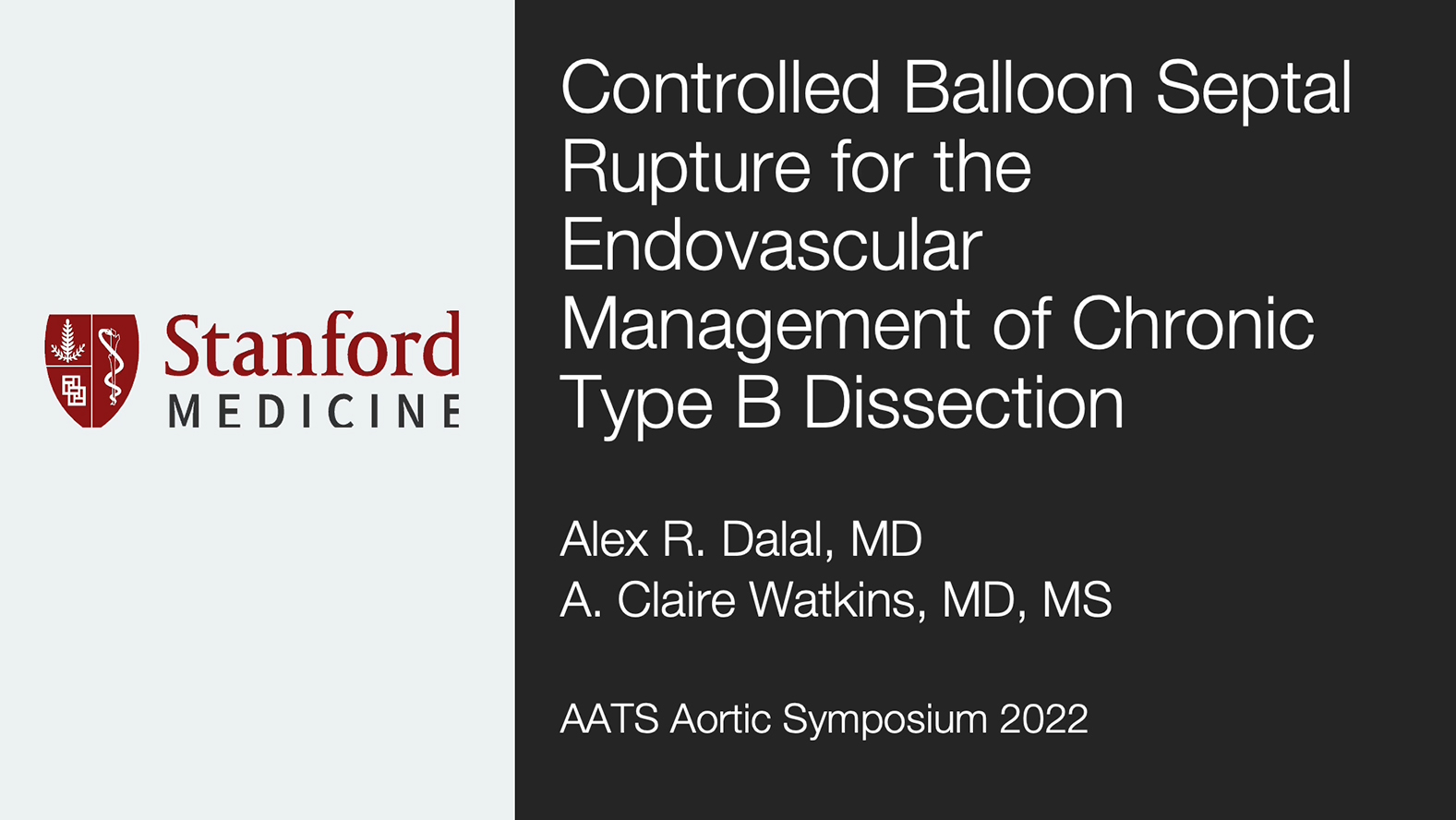

Supplement: Video 1 — A summary of the balloon septal rupture technique, results, and implications, including intraoperative examples. Video available at: https://www.jtcvs.org/article/S2666-2507(23)00037-8/fulltext. [file fx3.jpg]
